# No Kidney Left Behind: Rescuing Unused Donor Kidneys for Transplant at the First Centralized Assessment and Repair Center

**DOI:** 10.3389/ti.2025.15424

**Published:** 2025-11-28

**Authors:** Christopher L. Jaynes, William C. Goggins, Matthew L. Holzner, Jacqueline Garonzik-Wang, Henri G. D. Leuvenink

**Affiliations:** 1 Department of Surgery-Organ Donation and Transplantation, University Medical Center Groningen, Groningen, Netherlands; 2 34 Lives, Public Benefit Company, West Lafayette, IN, United States; 3 Division of Transplant Surgery, Department of Surgery, Indiana University School of Medicine, Indianapolis, IN, United States; 4 Recanti/Miller Transplantation Institute, Icahn School of Medicine at Mount Sinai, New York, NY, United States; 5 University of Wisconsin Department of Surgery, Madison, WI, United States

**Keywords:** normothermic perfusion, NMP, *ex vivo* kidney, ARC, SNAP

## Abstract

Rescue of non-used kidneys may be facilitated using sub-normothermic acellular machine perfusion (SNAP), which can prolong safe preservation times and provide additional viability assessment. While blood-based normothermic machine perfusion (NMP) has been utilized internationally, barriers to adoption of NMP in the US include limited availability, staffing and facility resource limitations, geographic distances between donor and recipient hospitals, blood shortages, and reliance on hypothermic machine perfusion (HMP). To overcome obstacles to adoption, the first centralized kidney Assessment and Repair Center (ARC) was established in West Lafayette, Indiana. Organized as an independent public benefit company, this center was designed to provide sub-normothermic acellular perfusion (SNAP) and assessment services to rescue unused/hard-to-place (HTP) kidneys. An acellular, human serum albumin-based perfusate was chosen, and a second cold ischemic time (CIT) was validated during pre-clinical testing. Between April 2024- May 2025, 158 unused deceased donor kidneys were transported to the ARC, resulting in 142 transplants (90%) after SNAP assessment. SNAP is feasible when performed by a centralized ARC and reduces kidney discard rates by providing objective viability assessment data. Moreover, SNAP allows for extended preservation times of nearly 70 h, enabling improved logistical planning and broader sharing of deceased donor kidneys.

## Graphical Abstract

**Figure:**
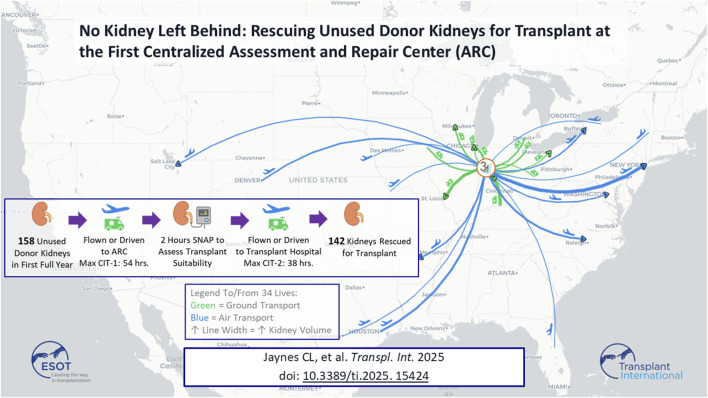

## Introduction

**FIGURE 1:**
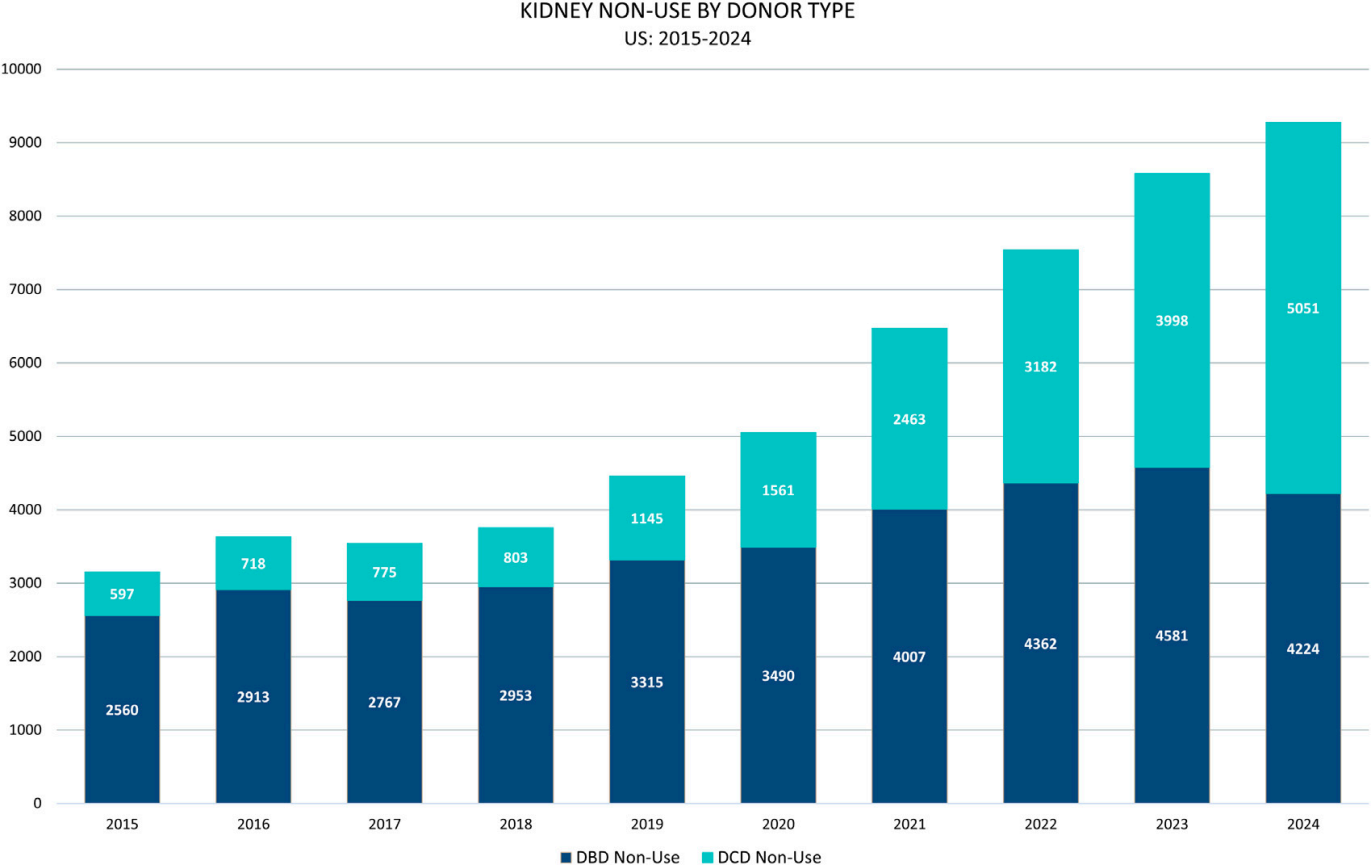
Kidneys recovered in the US with the intent to transplant but later not utilized (by donor type). DBD, donation after brain death; DCD, donation after cardiac death.

Kidney transplantation is a life-saving therapy for patients with end stage renal disease. As such, there are nearly 100,000 patients registered on the national kidney transplant waitlist in the US, with an average of 34 patients removed daily due to either dying or becoming too sick to receive a transplant. Due to the critical shortage of donor organs, kidneys are increasingly retrieved from non-ideal donors (older, more co-morbidities, etc.), leading to more uncertainty about organ quality, and resulting in escalating numbers of unused kidneys by the transplant centers (Figure 1) [1, 2].

Hard-to-place kidneys (HTP) have been previously defined as those accepted in the United Network for Organ Sharing (UNOS) match run after being refused at least 165 times by transplant centers. These kidneys have statistically higher donor risk profiles including higher rates of the following: Donor Age >50, Death by cerebrovascular accident (CVA), diabetes, hypertension, Donation after Circulatory Death (DCD), Kidney Donor Profile Index (KDPI) >85%, cold ischemia time (CIT) >24 h, terminal serum creatinine ≥1.5 mg/dL, and static cold storage preservation [3]. While many of the factors common to HTP kidneys cannot be changed (e.g., donor age, KDPI, cause of death), factors such as preservation technique and transportation time, are modifiable and may be able to decrease HTP kidney non-utilization rates.

In addition to addressing the modifiable factors, post-procurement functional assessment would help to alleviate uncertainties about HTP kidneys. Unfortunately, the depressed metabolic rate at hypothermic temperatures using static ice or hypothermic machine perfusion (HMP) preservation does not permit comprehensive organ assessment. The few assessment parameters available during HMP include internal renal resistance (IRR), pressure, and flow rate, which have been shown to have varied associations with kidney allograft function [4–6].

However, at temperatures approaching normothermia, kidney metabolism increases, and functional parameters can be assessed similarly as if the kidney was still *in vivo* in contrast to the other common preservation strategies (Table 1). The largest advancements in normothermic machine perfusion (NMP) over the past decade have been made for lung, heart, and liver perfusion, contributing to an increased use of these organs for transplantation by 50% or more [7–9]. However, the use of kidney NMP has been limited, in part because of its higher tolerance to cold ischemia, logistical barriers, and widespread adoption of HMP [10]. Currently, the published clinical NMP experience comes from international transplant centers entirely outside of the US. Due to the increased complexities required to maintain organs on NMP, it has been suggested that regional organ assessment and repair centers (ARCs) may be the most effective and efficient in providing this specialized modality [11]. Here, we present our early experience with the initiation and implementation of the first US ARC.

**TABLE 1:** Functional assessment data provided by kidney preservation type.

Comparison between kidney preservation methods			
Renal Biomarker	SCS (4 °C)	HMP (4 °C)	SNAP (35 °C)
Renal flow rate		✓	✓
Renal pressure		✓	✓
Renal resistance		✓	✓
Perfusate pO_2_/pCO_2_/pH			✓
Urine pO_2_			✓
TCO_2_/HCO_3_^-^/Base excess			✓
Perfusate Na^+^/K^+^/Cl^-^			✓
Urine Na^+^/K^+^/C^l-^			✓
Lactate/Glucose			✓
AST (marker of mitochondrial death)			✓
Oxygen consumption			✓
Hosgood transplant suitability score			✓

SCS, Static Cold Storage; HMP, Hypothermic Machine Perfusion; SNAP, Sub-normothermic Acellular Perfusion.

## Materials and Methods

### Facility

In 2022, a Public Benefit Company (“34 Lives, PBC”) was formed which built the first independent center in West Lafayette, Indiana, offering renal Sub-normothermic Acellular Perfusion (SNAP) assessment services to transplant hospitals and Organ Procurement Organizations (OPOs) to investigate the feasibility of an ARC in the US. This site was strategically chosen due to its proximity to Purdue University, a school with a strong reputation for engineering and an on-campus airport, but without a medical school or hospital, making it an ideal neutral location. In addition, the geographic location makes it possible to reach 80% of the US population within a 12-h drive, or 2.5-h flight.

### Perfusion Equipment

A two-room organ rescue laboratory (ORL) was constructed, in compliance with standard hospital operating room air exchanges and HEPA filtration, capable of providing SNAP to 4 kidneys simultaneously. The equipment platform has been described previously [12]. Briefly, a perfusion system was designed using a pediatric cardiopulmonary bypass circuit, consisting of a centrifugal pump (Bio-pump, Medtronic) and a membrane oxygenator with integrated heat exchanger (Affinity Pixie, Medtronic). The hardware included a speed controller, flow transducer, temperature probe (Bio-Console 560, Medtronic), and a heater/cooler (HCU30, Jostra).

### Sub-Normothermic Acellular Perfusion (SNAP) Protocol

After a thorough pre-clinical evaluation was carried out testing over 100 discarded human kidneys against several published NMP protocols [13–15], the SNAP protocol described by Minor *et al.* [16] was selected based on ease of use and repeatability of results. One of the major advantages of this protocol was the use of a non-blood-based, acellular perfusate, which is a safe alternative to erythrocyte-based perfusates at supraphysiological oxygen partial pressures [17]. Following the Minor protocol, the kidney is gently warmed from 8 °C to 35 °C using the oxygenated SNAP perfusate (composition, Table 2). At 90 min of SNAP, the kidney reaches its maximum temperature of 35 °C during which the first assessment occurs. A second assessment is completed after 120 min, allowing the observation of trends between timepoints. Kidneys were assessed for transplant suitability using a variety of validated and non-validated assessment parameters, including the Hosgood score [18], comprised of renal blood flow, urine output, and macroscopic appearance. Infrared thermal temperature measurements were taken to determine uniform perfusion, instead of relying solely on macro appearance, as a surrogate due to the clear acellular perfusate.

**TABLE 2:** Sub-normothermic Acellular Perfusion (SNAP) perfusate composition.

Snap perfusate ingredient	Amount
Calcium chloride dihydrate	0.33 g
Calcium gluconate	0.74 g
Cefazolin	1 g
D (+) glucose monohydrate	1.09 g
Dextran 40	5 g
Human albumin	70 g
Magnesium dichloride hexahydrate	0.12 g
Potassium chloride	0.54 g
Sodium bicarbonate	1.89 g
Sodium chloride	9.7 g
Sodium dihydrogen phosphate dihydrate	9.4 mg
Sodium hydroxide (1M)	pH 7.4
Verapamil	5 mg
Water for injection (WFI)	Fill to 2 L

### Clinical Kidney Rescue

**FIGURE 2:**
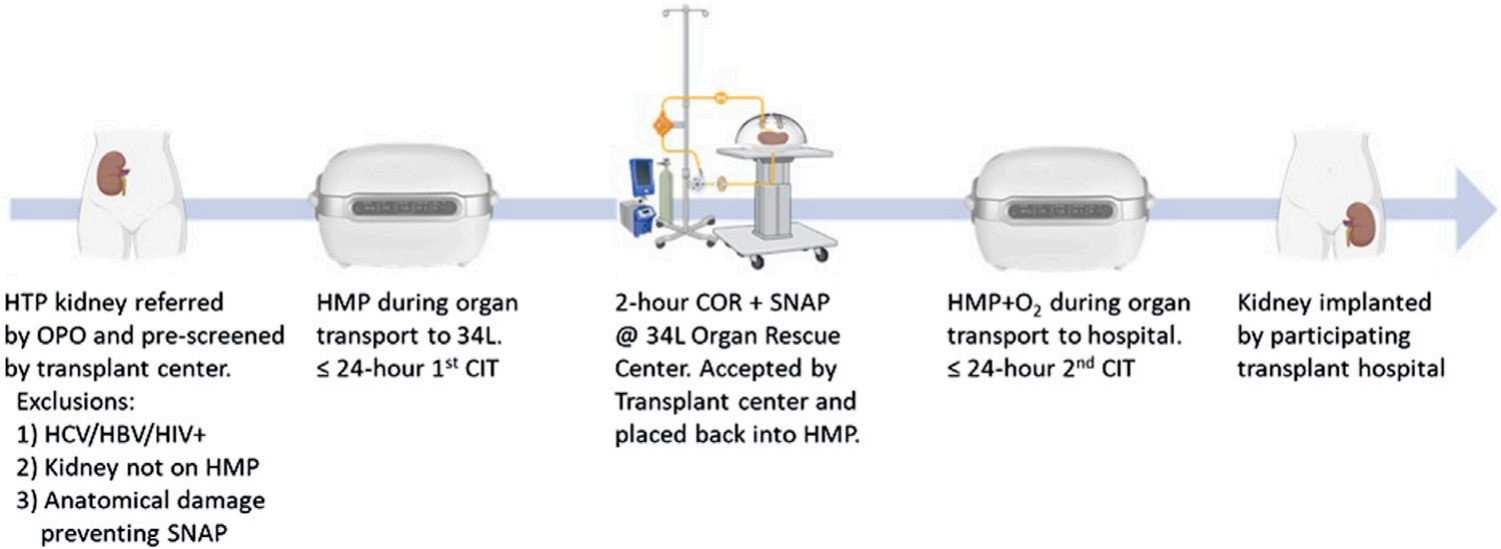
Optimal 34 Lives’ ARC workflow, beginning with OPO referral and completing with transplantation. 34L, 34 Lives; CIT, cold ischemic time; COR, controlled, oxygenated rewarming; HMP, hypothermic machine perfusion; HMP + O2, HMP with passive oxygen; HTP, hard-to-place; SNAP, Sub-normothermic acellular perfusion.

Clinical SNAP procedures began in April 2024, soon after the preclinical validation phase completed, and were allowed to proceed under the oversight of a Central Institutional Review Board (IRB). Three initial transplant hospitals (Indiana University Health, Mt. Sinai, and University of Wisconsin) piloted the centralized ARC service, ensuring both logistics and safety issues were appropriately considered. During this phase, HTP donor kidneys were offered to the ARC by OPOs after exhausting standard allocation efforts. Kidneys were subjected to pre-screening by the participating transplant program surgeons and nephrologists prior to accepting for SNAP assessment. Those with positive serologies (HIV, HCV, HBV), not on HMP, or with anatomical issues preventing SNAP were not evaluated. Beginning in October 2024, after the pilot phase, a total of 12 transplant hospitals, representing both large and small volume centers, participated and accepted SNAP-assessed kidneys through the remaining first year of ARC operation, ending in May 2025. After 2 hours of SNAP, kidneys with a Hosgood score of 1–3 (out of 5) were considered suitable for transplant, repackaged into a LifePort HMP device (ORS) using the passive bubble-oxygenated cassette pre-filled with Kidney Preservation Solution (KPS-1, ORS), and driven or flown by charter aircraft to the accepting transplant hospital (Figure 2). Each participating transplant hospital followed their own standard protocols for kidney implantation and immunosuppression. We hypothesized that sending HTP kidneys to a centralized ARC assessment facility utilizing peer-reviewed clinical SNAP protocols would result in a 50% or more success rate for allocation of these kidneys to a transplant center.

### Statistical Analysis

Descriptive statistics are presented as means, standard deviations, medians and ranges for the continuous variables and as frequencies and percents for categorical variables. A two-sided alpha level of 5% and 95% confidence intervals were used for all subsequent descriptive comparisons and analyses, unless otherwise stated. Demographics and baseline characteristics are summarized for all donors in the study group and overall point estimates and corresponding 95% confidence intervals. The study hypothesis was tested using a one-sample proportion z test to determine if the rate of allocation is different from 50%, with a 95% confidence interval constructed around the observed allocation rate. A p-value <0.05 is considered significant. GraphPad Prism version 10.5.0 software (La Jolla, California, USA) was used for analysis.

### Outcome Variables and Definitions

All outcome data was collected in a prospective manner. Donor variables were shared under a data use agreement by the participating OPOs via encrypted email and/or via DonorNet. CIT-1 is defined as the time from donor cross clamp to the beginning of SNAP. CIT-2 begins after SNAP stops until reperfusion in the recipient. Total out of body time starts at donor cross clamp and ends at recipient reperfusion. Additional variables collected at 90 (T90) and 120 (T120) minutes included arterial and venous blood gas samples, urine output (UO), renal blood flow (RBF) intrarenal resistance (IRR), aspartate aminotransferase (AST) as a marker of mitochondrial dysfunction [19], and macroscopic kidney appearance (pink, patchy, mottled).

## Results

### HTP Kidney Acceptance

**FIGURE 3:**
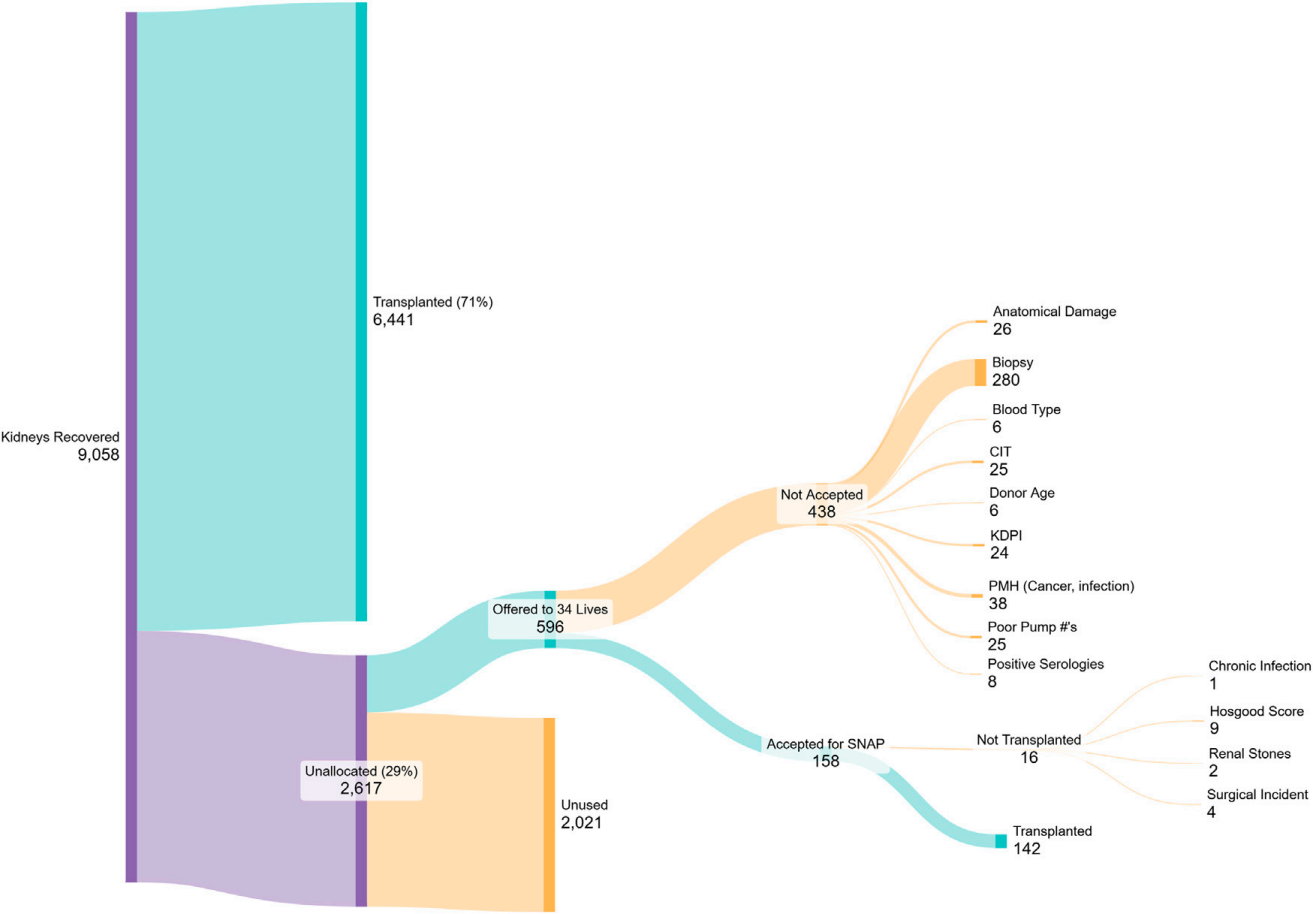
Sankey diagram beginning on the left with kidneys recovered from the referring OPOs during the first year of 34 Lives’ ARC operations. 71% of recovered kidneys were transplanted from these OPOs, while 29% went unused. Of those, 142 were transplanted after SNAP, while the others were not accepted for transplant based on the common reasons shown.

Out of 158 HTP donor kidneys accepted and assessed with SNAP during the first full year, 142 (90%) were determined suitable by the accepting transplant physician and transplanted, surpassing the primary hypothesis of a 50% success rate. The median sequence number on the UNOS waiting list for patients receiving a SNAP rescued kidney was 3,302 (mean 5,164), further confirming that these kidneys were HTP. Reasons for non-acceptance and non-use can be found in Figure 3.

### Donor Characteristics

Donor Characteristics are summarized in Table 3. Of note, more donors were females (56%), donating after circulatory death (DCD, 62%) due to anoxia (58%), with past medical histories including hypertension (61%), smoking (74%), and a median KDPI of 71% (range 19%–98%).

**TABLE 3:** Donor characteristics (n = 142).

Age (median years, range)	53 (15–73)
Sex (n, %)	
Males	62 (44%)
Females	80 (56%)
BMI (median kg/m^2^, range)	30.63 (17.7–58.5)
Kidney laterality (n, %)	
Left	67 (47%)
Right	75 (53%)
Donor type (n, %)	
DBD	54 (38%)
DCD (no NRP)	57 (40%)
DCD + NRP	31 (22%)
Comorbidities (n, %)	
HTN	86 (61%)
Diabetes: A1C ≥6.5%	19 (13%)
Smoker	105 (74%)
Cause of death (n, %)	
Anoxia	82 (58%)
CVA/ICH	38 (27%)
Head trauma	19 (13%)
Other	3 (2%)
Inotropes (n, %)	
0 vasopressors	32 (22%)
1 vasopressor	110 (77%)
2 vasopressors	61 (43%)
3+ vasopressors	20 (14%)
Biopsies	
Glomerular sclerosis (median %, range)	7 (0–71)
Vascular changes (mild: 10%–24%)	39 (32.5%)
Vascular changes (moderate: 25%–50%)	2 (1.7%)
ATN (n, %)	13 (11%)
Creatinine (mg/dL; median, range)	
Peak	1.91 (0.5–11.09)
Terminal	1.24 (0.4–8.6)
KDPI (median %, range)	71 (19–98)

Unless otherwise specified, variables are expressed as actual counts. A1C, glycated hemoglobin; ATN, acute tubular necrosis; BMI, body mass index; DBD, donation after brain death; DCD, donation after cardiac death; CIT, cold ischemic time; CVA, cerebral vascular accident; ICH, intra-cerebral hemorrhage; HTN, hypertension; KDPI, kidney donor profile index; NRP, normothermic regional perfusion.

### HMP and SNAP Characteristics

#### Total Preservation Time

**FIGURE 4:**
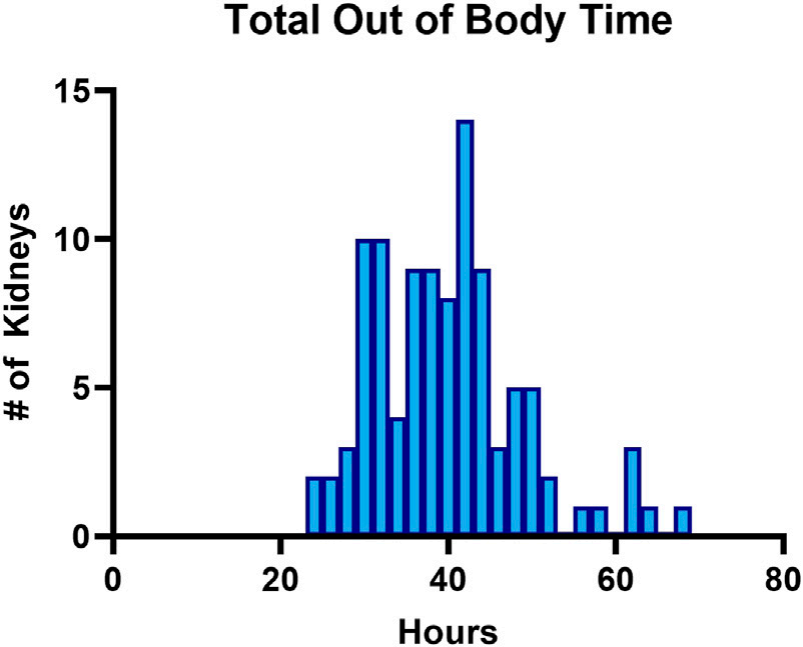
Frequency distribution showing total times (in hours) of how long kidneys were preserved using a combination of HMP and SNAP outside of the donor prior to implanting into a recipient.

In this protocol, there are two distinct periods of CIT which flank the SNAP assessment period. The first CIT (CIT-1) starts at donor cross clamp and ends when SNAP begins. Median CIT-1 was 21.15 h (range 10.45–54.37 h) prior to SNAP. CIT-2 starts when the kidney begins cool-down after SNAP for a second round of HMP and ends at kidney reperfusion in the recipient. Median CIT-2 was 14.5 h (range 2.87–37.75 h). The median time that HTP kidneys were preserved *ex vivo* using a combination of HMP and SNAP was 39.78 h (range 23–67.78 h). Figure 4 depicts the distribution frequency of total out-of-body preservation times.

#### HMP Changes

The median IRR during HMP and prior to SNAP was 0.25 (range 0.07–1.03) with median flow rates of 106 mL/min (range 33–185). After SNAP, the compliance improved significantly (IRR 0.15, *p < 0.0001)*.

#### SNAP Changes

**FIGURE 5:**
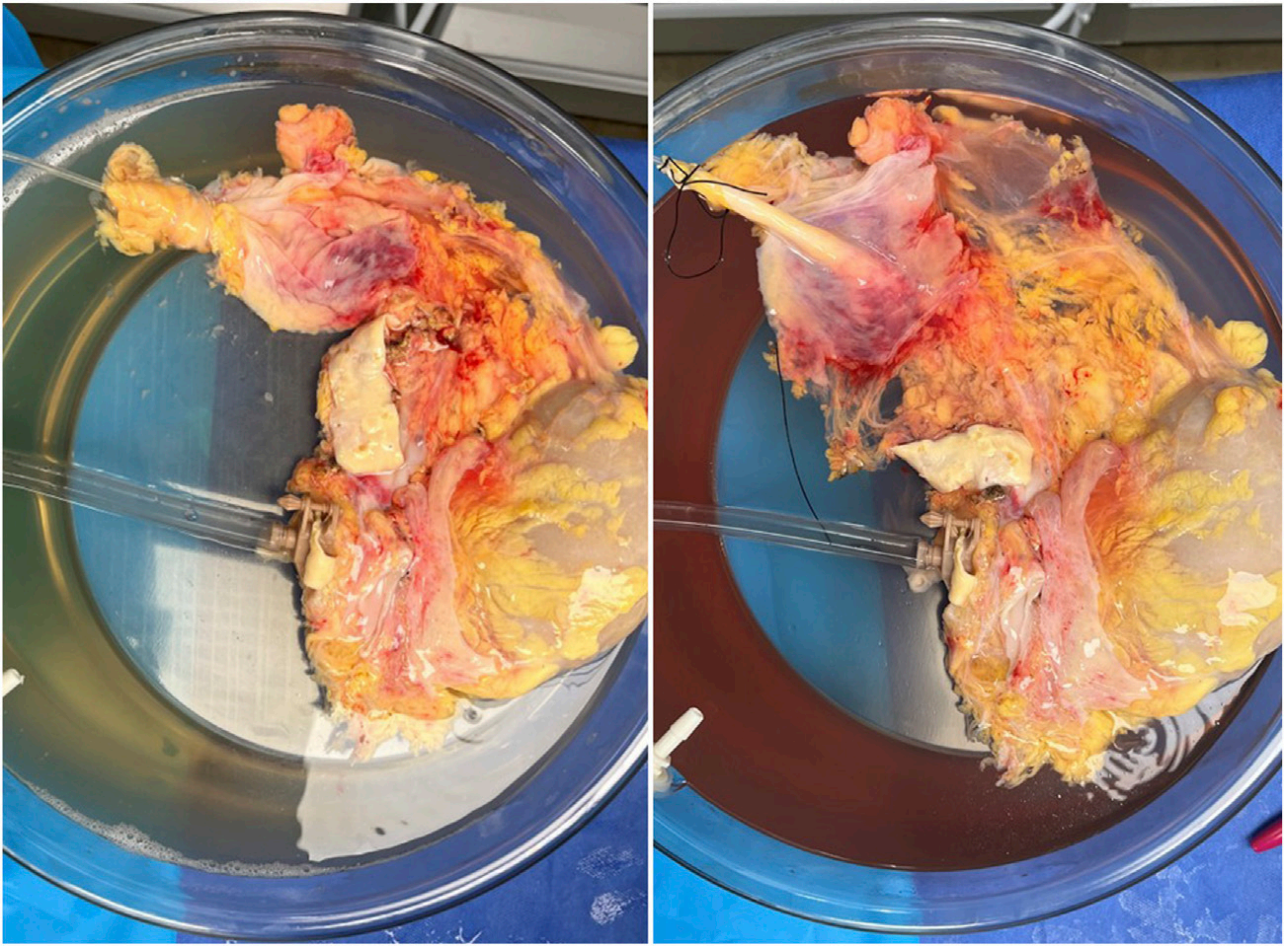
Typical donor kidney at start (left) and end (right) of SNAP. As the kidney warms past 30 °C, the perfusate color turns from amber to red as the vasculature becomes more compliant.

Once the temperature reached 30 °C, the kidney vasculature started to release a visible red effluent as shown in Figure 5. This phenomenon occurred in all HTP kidneys assessed during SNAP, despite a standard donor flush with UW or HTK solution and transport of up to 54 h on an HMP device. Table 4 summarizes the assessment characteristics used to determine transplant suitability. Statistically significant (*p < 0.0001)* improvement in median assessment parameters at 90- and 120-min during SNAP includes flow rate (20% higher), IRR (15% lower), urine output (115% higher), oxygen consumption (16% higher), and mean Hosgood Score (14% lower). Statistically significant functional changes in median glucose (3% drop), lactate (120% increase), AST (19% increase) and weight gain (3% increase, *p = 0.0009*) were also observed, but did not thwart transplantation.

**TABLE 4:** HMP and SNAP characteristics (n = 142).

	Pre-SNAP	Post-SNAP	% Change	pValue	
HMP flow rate (mL/min)	106 (33–185)	103 (33–229)	−6%	0.42	ns
HMP pressure (mmHg)	32 (13–49)	20 (10–40)	−38%	<0.0001	****
HMP IRR (mmHg/mL/min)	0.25 (0.07–1.03)	0.15 (0.05–0.78)	−39%	<0.0001	****
Kidney weight (g)	478 (241–1,062)	493 (242–1,180)	3%	0.0009	***

	SNAP 90 min	SNAP 120 min	% Change	pValue	
SNAP flow rate: (mL/min)	560 (170–1,360)	670 (220–1,580)	20%	<0.0001	****
SNAP pressure (mmHg)	75 (75–75)	75 (75–75)	0%	1	ns
SNAP IRR (mmHg/mL/min)	0.13 (0.06–0.44)	0.11 (0.05–0.34)	−15%	<0.0001	****
Urine output (mL/hour)	13.5 (0–234)	29 (0–315)	115%	<0.0001	****
Arterial pO2 (mmHg)	570.2 (514–705)	571 (504.5–720)	−1%	0.0005	***
Venous pO2 (mmHg)	393 (120–594)	412.8 (210–587)	5%	0.0004	***
Venous pCO2 (mmHg)	43.5 (23–58.8)	42.2 (23–55.6)	−3%	<0.0001	****
Perfusate sodium (mEq/L)	142 (104–151)	144 (137–158)	1%	<0.0001	****
Perfusate potassium (mmol/L)	6.1 (4.3–12)	5.9 (4.1–12)	−3%	<0.3384	ns
Perfusate chloride (mEq/L)	116.5 (111–131)	117 (111–139)	0.4%	0.1508	ns
Perfusate glucose (mmol/L)	113 (74–164)	110 (73–153)	−3%	<0.0001	****
Perfusate lactate (mmol/L)	0.68 (0.3–4.1)	1.5 (0.3–4.24)	120%	<0.0001	****
Perfusate AST (U/L)	26 (9–278)	31 (10–340)	19%	<0.0001	****
Urine pO2 (mmHg)	183.1 (83–284)	190.4 (117–259)	4%	0.8246	ns
Urine sodium (mEq/L)	130 (85–153)	129 (83.5–147)	−0.8%	0.0252	*
Urine potassium (mmol/L)	10.64 (3.5–19.96)	11.3 (5.1–20)	6%	0.0961	ns
Urine chloride (mEq/L)	108.5 (65–123)	108.9 (60–128.6)	0.4%	0.1519	ns
Oxygen consumption (mLO_2_/(min*125 cm [3]))	2.2 (0.45–6.25)	2.55 (0.75–6.55)	16%	<0.0001	****
Hosgood score (1–5)	2 (1–3)	2 (1–3)	0%	<0.0001	****
CIT-1 cross clamp to start of SNAP (hours, range)			21.15 (10.45–54.37)		
CIT-2 end of SNAP to recipient reperfusion (hours, range)			14.5 (2.87–37.75)		
Total out of body time (hours, range)			39.78 (23–67.78)		

Unless otherwise specified, variables are expressed as medians. AST, aspartate aminotransferase; CIT, cold ischemic time; IRR, intrarenal resistance; HMP, hypothermic machine perfusion; pO2, partial pressure of oxygen; SNAP, sub-normothermic acellular perfusion.

### Centralized ARC Transportation Logistics

HTP kidneys accepted for SNAP with ≤6-h ground times to the 34 Lives’ ARC were generally driven by medical couriers on LifePort HMP devices. If >6-h ground times were necessary, kidneys were typically flown by charter aircraft due to the commercial airline restrictions for liquids and lithium-ion batteries, common to HMP devices. During the first full year, 37 (26%) of the HTP kidneys were flown a total of 35,579 miles to the 34 Lives’ ARC from the originating OPOs, while 105 (74%) were driven a combined 22,622 miles. Similarly, from the 34 Lives’ ARC to the accepting transplant center, ground times ≤6-h were driven and >6-h were flown by charter aircraft to minimize CIT-2. During this time, 77 (54%) of the SNAP kidneys determined suitable for transplantation were flown from the 34 Lives’ ARC to the accepting transplant centers for a total of 54,698 miles, while 65 (46%) were driven a combined 12,409 miles. One kidney in this study had a 6-h delay arriving at the 34 Lives’ ARC due to the driver hitting a deer *en route*, but the kidney arrived safely and was successfully utilized after SNAP.

## Discussion

Novel solutions are needed to address the escalating US kidney discard rate to ensure that suitable kidneys are safely transplanted. While many unused kidneys cannot be rescued due to anatomical issues and donor histories (e.g., uncontrolled HTN, diabetes, cancers, infections), a meaningful number can be saved for transplantation following SNAP assessment. Our centralized ARC facilitated transplantation of 142/158 (90%) unused/HTP kidneys after SNAP during the first year of clinical operation, surpassing the initial 50% success rate hypothesis. This demonstrates feasibility and the promise for increased access to kidneys for thousands of patients awaiting transplantation.

In 2017, the National Kidney Foundation (NKF) convened a meeting of key opinion leaders with diverse backgrounds including representatives from NKF, OPOs, transplant centers, the Centers for Medicare and Medicaid Services (CMS), the Health Resources and Services Administration (HRSA), the National Institutes of Health (NIH), the Scientific Registry of Transplant Recipients (SRTR), UNOS, and private health insurers. The goal of the conference was to develop actionable recommendations from key stakeholders to increase the use of more HTP kidneys and to decrease discard rates. The group made several recommendations [20], shown in Table 5, along with how each can be addressed by a centralized ARC.

**TABLE 5:** NKF consensus group recommendations to decrease kidney discard rates.

Consensus recommendation	34 Lives’ central ARC benefit
Create expedited placement pathways to directly offer organs at risk of discard to a small subset of centers that opt in to accept these organs	HTP kidneys offered by OPOs are assessed and accepted by participating transplant centers, increasing allocation success
Identify organs at risk of discard during standard allocation and shunt them to patients at transplant ‘rescue’ centers that utilize high-risk organs when standard placement is unsuccessful	HTP kidneys are offered sooner to the participating transplant ‘rescue’ centers when standard allocation efforts are unsuccessful, increasing odds of transplant
Standardize provision of gross photographs of procured kidneys to share on DonorNet	The 34 lives’ central ARC provides photographs, videos, and digitized biopsy slides, highlighting kidney vasculature and including a ruler in the picture to help the surgeon determine scale
Develop decision and support tools to help surgeons evaluate the benefits and downstream risks of accepting or refusing an organ	The 34 lives’ central ARC provides several additional assessment tools, enabling the transplant center to better evaluate kidney function data not currently available with static ice storage or HMP.
Optimize kidney recovery and management, including expanding research opportunities on normothermic *ex-vivo* perfusion to recondition organs from older donors or those with long cold ischemic times	The use of SNAP for HTP kidneys provides a standard platform for assessment of marginal organs due to extended CITs, poor initial biopsies, or from older donors and will provide new tools to assess advanced therapeutics and repair efforts in the future

SNAP provides more assessment data, including Hosgood Score, IRR, oxygen consumption and urine output, than is possible from static ice or HMP preservation, giving HTP kidneys extended time for a second look by transplant physicians. Using SNAP, we were able to preserve kidneys *ex vivo* for up to 68 h, which is significantly longer than the recommended standard of care of <24 h [21, 22]. This extended time allowed many transplant centers, including several that rejected the initial OPO offer for the same kidney, but later accepted after reviewing SNAP assessment data, to safely utilize these kidneys. While initial SNAP rescue rates seem promising, additional data will be necessary to verify the sustainability of SNAP provided by a centralized ARC and will be reported once long-term patient and graft survival data have been collected and analyzed.

While SNAP provides new tools to assess kidney function prior to transplantation, there still is much work to be done to decrease the rates of kidney non-use. From the Sankey diagram in Figure 3, the biggest potential improvement comes from encouraging OPOs to refer more HTP kidneys to the ARC (only 23% of the unallocated kidneys were referred to the ARC in the first full year from the participating OPOs). Additionally, decreasing the rate of kidneys not accepted for SNAP should include developing better assessment tools that discourage the current reliance on renal biopsy, KDPI and poor HMP pump numbers, and instead encourages trust in the functional assessment provided by SNAP.

SNAP is feasible when performed by an independent, centralized ARC and reduces kidney non-use rates by providing objective viability assessment data, enabling up to a 90% success rate in transplant allocation. Transporting unused/HTP donor kidneys to a dedicated ARC provides transplant hospitals with additional time to locate and screen potential recipients and additional data to make better informed decisions not otherwise available with static ice or HMP preservation methods. Moreover, SNAP allows extended preservation times close to 70 h, enabling improved logistical planning and broader sharing of deceased donor kidneys. Increasing utilization of the current pool of available human donor kidneys is possible, offering an immediate solution to the current donor organ shortage, while providing significant and immediate benefits.

## Data Availability

The raw data supporting the conclusions of this article will be made available by the authors, without undue reservation.

## Author’s Note

These authors were responsible for the initial study conception, contributed to the article, served as the initial clinical investigators, provided data used in the submission, and approved the submitted version.
